# Health sector reforms and human resources for health in Uganda and Bangladesh: mechanisms of effect

**DOI:** 10.1186/1478-4491-5-3

**Published:** 2007-02-01

**Authors:** Freddie Ssengooba, Syed Azizur Rahman, Charles Hongoro, Elizeus Rutebemberwa, Ahmed Mustafa, Tara Kielmann, Barbara McPake

**Affiliations:** 1 Health Policy, Planning & Management, Makerere University, Institute of Public Health, Republic of Uganda; 2 Department of Public Health and Policy, Health Policy Unit, London School of Hygiene and Tropical Medicine, Keppel Street, London WC1E 7HT, United Kingdom of Great Britain and Northern Ireland; 3 Health Systems Trust, 1st Floor Riverside Centre, Belmont & Main Road, Rondebosch, 7700, Republic of South Africa; 4 Ministry of Health and Family Welfare, Dhaka, People's Republic of Bangladesh; 5 Institute for International Health and Development, Queen Margaret University College, Corstorphine, EH12 8TS, United Kingdom of Great Britain and Northern Ireland

## Abstract

**Background:**

Despite the expanding literature on how reforms may affect health workers and which reactions they may provoke, little research has been conducted on the mechanisms of effect through which health sector reforms either promote or discourage health worker performance. This paper seeks to trace these mechanisms and examines the contextual framework of reform objectives in Uganda and Bangladesh, and health workers' responses to the changes in their working environments by taking a 'realistic evaluation' approach.

**Methods:**

The study findings were generated by triangulating both qualitative and quantitative methods of data collection and analysis among policy technocrats, health managers and groups of health providers. Quantitative surveys were conducted with over 700 individual health workers in both Bangladesh and Uganda and supplemented with qualitative data obtained from focus group discussions and key interviews with professional cadres, health managers and key institutions involved in the design, implementation and evaluation of the reforms of interest.

**Results:**

The reforms in both countries affected the workforce through various mechanisms. In Bangladesh, the effects of the unification efforts resulted in a power struggle and general mistrust between the two former workforce tracts, family planning and health. However positive effects of the reforms were felt regarding the changes in payment schemes. Ugandan findings show how the workforce responded to a strong and rapidly implemented system of decentralisation where the power of new local authorities was influenced by resource constraints and nepotism in recruitment. On the other hand, closer ties to local authorities provided the opportunity to gain insight into the operational constraints originating from higher levels that health staff were dealing with.

**Conclusion:**

Findings from the study suggest that a) reform planners should use the proposed dynamic responses model to help design reform objectives that encourage positive responses among health workers b) the role of context has been underestimated and it is necessary to address broader systemic problems before initiating reform processes, c) reform programs need to incorporate active implementation research systems to learn the contextual dynamics and responses as well as have inbuilt program capacity for corrective measures d) health workers are key stakeholders in any reform process and should participate at all stages and e) some effects of reforms on the health workforce operate indirectly through levels of satisfaction voiced by communities utilising the services.

## Background

In the last two decades, developing country governments have implemented a variety of reforms in the health sector on the understanding that these reforms would create the right individual and organisational incentives for improving health systems performance. However, reform initiatives have not always considered human resource issues that are relevant to their success and have often failed to include the participation or perspectives of the health workforce in reform planning processes and decision-making.

A number of studies have considered the effects of reforms on the health workforce [[1-5] and [6]] and highlight the importance of human resources to the success of reform objectives [7] as well as the complexity of human resource management in the context of reforms [8,9].

These studies have pointed out that human resource issues need to be a primary consideration in reform design, suggesting that reforms can only be implemented successfully where there is consensual participation on the part of the workforce. Ngufor describes how health staff in Cameroon perceived reforms as a punishment inflicted on the nation by the International Monetary Fund (IMF) and the World Bank and as a result developed a laissez faire attitude to their work resulting in reduction of consultation times and absenteeism [4]. Similarly, workers in Zimbabwe were reported to perceive reforms as threatening their job security, salaries and training and expressed their demotivation in the form of unethical behaviour with their patients and neglect of work responsibilities [6]. In other parts of the world, health workers have resisted change on the grounds of conflicting values. In Latin America, for example, reforms were perceived as aiming to undermine the fundamental values that had inspired the design of the system, and sparked off a wave of resistance and strikes in El Salvador and Mexico. This led to the stalling and delay of the reform process [10,11].

Other studies have noted higher motivation levels among the health workforce through reforms. In Kazakhstan, reforms that aimed at changing the old Soviet system through the introduction of more market-oriented financing and service delivery, were argued to have resulted in increased interest in primary care among physicians, increased attention to quality and patient satisfaction, more rational and creative use of resources, and stronger commitment of physicians' personal time and resources to improve services for patients [12]

Despite the expanding literature on how reforms may affect health workers and which reactions they may provoke, little research has been conducted on the mechanisms through which health sector reforms either promote or discourage health worker performance. How do reforms affect the health workforce and create responses that are likely to encourage the success or failure of reform objectives? How does context influence the routes through which reforms affect provider incentive environments and eventually motivation and performance? To address these questions, the following paper seeks to trace these mechanisms of effect and examines health workers' responses to the changes in their working environments by taking a 'realistic evaluation' approach [13].

**Figure 1:**
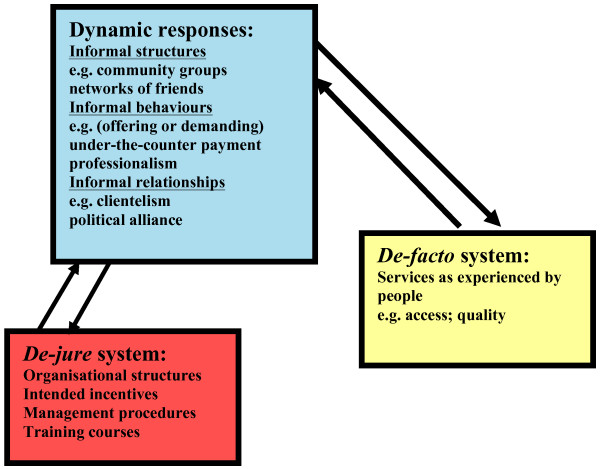
A conceptual model for health systems research – the Dynamic Responses Model [14].

This approach takes account of the explanatory mechanisms and the context of health systems reforms. This stands in contrast to the more common evaluation approach, which tends to focus on a reform programme's measures and its intended effects and then seeks to measure the differences between reforming and non-reforming entities, within the dimensions of those intended effects. The research described above suggests that reform programmes cause a multitude of workforce responses which act as the lynchpin between formal arrangements at the outset of the reforms and the resulting changes in the system as experienced by people who use it. Figure 1, originally created by McPake [14], captures the essence of the scenario described above and provides a framework for health systems research.

The focus of enquiry lies within the three components outlined above. While the de-jure system sets the context and defines the organisational structures, the intended incentives and management procedures, the dynamic responses reflect how those implementing the de-jure system respond and create 'informal' arrangements or behaviours that may or may not encourage the success of 'formal' reform objectives initiated by the de-jure system. The de-facto system then encompasses the lived experience of the results of the relationship between the de-jure system and the dynamic responses. Similar to realistic evaluation, the model emphasises the mechanisms of effect between a social programme (component of the de-jure system), and its impact (in the de-facto system), and recognises these mechanisms as being social: involving the interaction of human beings in a particular context that determines the nature and form of dynamic responses [14].

In this paper, we seek to focus on the mechanisms through which unintended human resource effects have arisen, using the case studies of Bangladesh and Uganda, two countries that have faced many reforms attempting to improve health systems performance and delivery of care. Both countries have extremely low per capita expenditure on health (Bangladesh has a public health expenditure of 14 international dollars, one of the lowest in the region and Uganda's public health expenditure lies at 22 international dollars) and face challenges in the attainment of the Millennium Development Goals and national development. After 20 years of military regime, Bangladesh became a parliamentary democracy in 1991. With rigid central government structures and disagreement between the main political parties inhibiting response to local health needs, the country began a wide programme of reforms to address issues of responsiveness. In Uganda, a series of reforms were introduced from the early 1990s as part of a larger initiative to restore the health system following its collapse during the political crises of the 1970s.

Focusing on selected reforms in each country (see Table 1) the first section of the paper outlines the scope of the reforms implemented and provides observations on workforce responses made by earlier evaluations. The subsequent section then presents the results of this research, exploring the impact of the mechanisms on social interactions.

**Table 1:** Selected reform initiatives in Uganda and Bangladesh

Uganda	Bangladesh
• Decentralisation of governance to district councils, 1993	• Unification of health and family planning services at sub-district level, 1998 – 2003
• Civil service reforms, 1990 onwards	∘ Procurement
∘ Pay reforms	• Training of health and family planning personnel at sub-district level, 1998 – 2003
• User fees implementation in 1992 and retraction in 2001	• Introduction of clinics at the community level, 1998 – 2000
• Health sub-district policy, 1999	

## Methods

The paper is based on a study conducted in 2004 examining mechanisms of effect through which selected health sector reforms have impacted on human resources in the national contexts of Bangladesh and Uganda. The study had the following main objectives:

1. To critically analyse health sector reforms in relation to the macro-level environment (policy level analysis: financing, regulation, organisation and management): or to understand the contextual de-jure system.

2. To examine the pathways through which selected reforms have impacted on the incentive environment for health workers (authority and accountability structures, career structures, staff recruitment, payment) and the responses they have produced: dynamic responses

### Study setting and design

The study was undertaken in six district zones in each country. The study districts in both countries were identified using a socio-economic stratification method employing the Human Development Index (HDI) ranking. The HDI takes three main indicators into account: life expectancy, educational attainment as a proxy measure of literacy and GDP per capita as a measure of standard of living. Together, these indicators show the level of need and the capacity of districts to benefit from health intervention [15]. The method was chosen to capture a wide range of reform experiences across varying socio-economic development levels. It was expected that recruitment patterns, survival activities and community responses might operate differently in districts of varying prosperity levels, and that sampling across the development level range would increase the breadth of experience likely to be captured. Study districts were randomly selected from three equal strata ranked by HDI, that is, two districts were selected from each stratum. Tables 2 and 3 show the selected districts and their HDI ranking in Uganda and Bangladesh respectively.

**Table 2:** Uganda: Human Development Index of selected study district

Study district	HDI	Group rank
Kampala	0.593	High
Jinja	0.534	High
Bushenyi	0.456	Average
Mubende	0.458	Average
Moyo	0.361	Low
Arua	0.383	Low
Lira	0.405	Average

**Table 3:** Bangladesh: Human Development Index of selected study district

Study district	HDI	Group rank
Jamalpur	≥0.501	High
Thakurgaon	≥0.501	High
Cox's Bazar	0.401-0501	Average
Moulvibazar	0.401-050	Average
Barguna	<0.401	Low
Chuadanga	<0.401	Low
Sirajgonj	0.451–0.501	Average

### Methods of data collection and analysis

The information for the study was generated initially by reviewing published and government documents and then by triangulating both qualitative and quantitative methods of data collection and analysis among policy technocrats, health managers and groups of health providers. A comparison was made between providers in the family planning and health tracks in Bangladesh and between public and non-governmental organisation (NGO) sectors in Uganda. The rationale for the comparative groups was based on the nature of the major reforms and the human resource groups that they targeted.

**Documents **– published and government reports from both countries – were analysed in the formative phase of the study which was undertaken with the purpose of understanding the reform processes and focusing on their objectives, design, implementation and structural impacts on health workers at national, district and health facility levels.

**District level surveys **of health workers were conducted between February and June 2004. In Bangladesh, 703 providers were interviewed individually, while, in Uganda, the total number of providers interviewed amounted to 800 (see Table 4 for breakdown of interviews per district). A questionnaire that had been pilot-tested and translated, where necessary, elicited health workers' perceptions of the effects of the selected reforms on their organisation and on themselves as workers. Scaled responses were used to gauge perceptions of the following characteristics of their job: management authority, accountability, career development, recruitment pattern of staff and their deployment, payment schedule, promotion opportunity and survival strategy. Health workers could respond to the close-ended questions with 'Strongly agree', 'Partially agree', 'Do not agree' and 'Do not know', which allowed a wider scale of responses and captured the middle ground. In Uganda, all the staff working in the health centres at the study sites were interviewed and an attempt was made to interview at least 50% of doctors and clinical assistants and 20% of nurses at the selected hospitals. Due to erratic staff availability and attendance on survey days, these guidelines were only partially followed. In Bangladesh, a sample of health workers was taken from each of the four different types of public health facilities located at district, sub district and community levels. Clinical workers such as medical assistants and family welfare visitors were selected from the community level.

**Table 4:** Number of provider interviews by districts

**Uganda**		**Bangladesh**	
District	Number of respondents	District	Number of respondents

Kampala	199	Jamalpur	121
Jinja	122	Cox's Bazar	115
Bushenyi	120	Moulvibazar	119
Mubende	139	Barguna	113
Moyo	102	Chuadanga	117
Arua	118	Gaibandha	118

The quantitative data from both countries were analysed using SPSS and presented in frequency tables.

**Key Informant Interviews **were conducted with district level health managers and officials in the central government ministries responsible for health, public service and local government. The interviews focused on the context, the content and the process of reforms and elicited perceptions of how reforms affected ground-level realities within health facilities and the workforce. In Uganda the interviews were conducted by social science graduates who had prior experience in qualitative data collection. After two days of orientation regarding the study objectives and methods, a semi-structured interview guide developed by the core research team was piloted and then utilised to conduct the interviews. The guide aimed to explore the main domains of the reforms and their possible effects on human resources. The findings from these interviews were used to identify the themes and questions for the individual staff survey. In Bangladesh, questions for the key interviews were drafted and discussed with the Health Systems Development Programme office and conducted by the principal and co-investigators at the national level and by a trained research associate at the district level. National level key informants were interviewed about the reform processes and implementation issues, while district level informants were asked to respond to service delivery issues, practical experiences and constraints at ground-level, as well as issues concerning the coordination and cooperation of the two cadres (health and family planning) in Bangladesh, their incentives, workload and job environment.

**Focus Group Discussions **were conducted with different cadres of health workers (nurses, midwifes and clinical officers and associated field cadres) to explore how the roles and support of the organisations concerning their human resource management functions changed under the reforms. In Uganda six focus group discussions were held in Lira. The participants were grouped according to their cadres i.e. midwives (three groups), nurses (three groups) and clinical officers and allied health workers (two groups). Members from each group were invited from 2–5 health facilities to Lira, where the discussions were held. The individual participants were selected from a list of district personnel provided by the District Records Officer. In Bangladesh, eight focus group discussions were held in Sirajgonj. Here again the participants were grouped according to their cadres (Upazila Family Planning Officers (UFPO) and members of the Ministry of Mother and Child Health (MOMCH); Upazila Health and Family Planning Officers (UHFPO); medical doctors from the district hospitals; senior staff nurses from the district hospitals; senior staff nurses from the Upazila Health Complex; paramedical staff; Sub Assistant Community Medical Officers (SACMO) and family welfare visitors (FWV); family welfare assistants (FWA) and family planning inspectors (FPI)) and discussions took place at the district hospital and Upazila health complexes. Upazila refers to the Health Sub-district level in the Bangladeshi health system. Guidelines for the topics were prepared and discussions were conducted by a co-ordinator of the study together with three trained research associates.

The qualitative information was compiled and analysed following standard qualitative data analysis techniques with master sheets enabling the identification of emerging themes and quotes that illustrated the thematic issues.

## Results

### The context of reforms and their effects on intended objectives: de-jure system

#### Bangladesh

After partition from India in 1947, Bangladesh achieved full independence in 1971 and became a parliamentary democracy in 1991 after 20 years of military regime. With rigid central government structures and disagreement between main parties largely inhibiting response to local health needs [16], Bangladesh began a wide programme of reforms to address issues of responsiveness. The main reforms in Bangladesh aimed at integrating the two separate divisions of health services and family planning thus unifying the two programmes with the intention of improving their efficiency and responsiveness to the user population.

##### Unification

The process of unifying service structures started in 1998 at the sub-district (*Upazila*) level and below with the intention of gradually, and eventually, unifying overall management. Unification entailed reorganisation and restructuring of health and family planning delivery systems to ensure a common management structure. All health and family planning activities at the Upazila level were placed under the unified authority of the UHFPO, who is a medical doctor by profession and formerly belonged to the health directorate as a cadre. Family planning workers were moved to a formal civil service (recurrent budget) payroll. This shift had implications for financial management, performance appraisal and workloads.

Historically, health and family planning programmes had separate budgets and the drawing and disbursement of funds were handled separately for each track. After unification, the financial authority to draw and disburse funds at the Upazila level was delegated to three officers: the Upazila Health and Family Planning Officer in overall charge, the Upazila Family Planning Officer and the Maternal and Child Health Family Planning Officer.

The unification process reassigned the role of performance appraisal for both the management team at the Upazila level and for the operational staff at lower levels. The major change in the appraisal system was that individuals from a different professional or programme background were put in charge of appraising personnel [17].

The integration of family planning and health services brought a new set of tasks to be carried out by all frontline personnel. Thirty-seven tasks were identified as components of the health worker's job description at the community clinics [18]. Most of these new tasks were added on to the work of family planning workers while a comparatively small set of tasks was assigned to workers of the Health Directorate.

##### Procurement

The reforms in procurement and logistics management created a centralised procurement with guidelines necessitating a multistage procedure. The separate logistical management systems of health and family planning services prior to the Health and Population Service Program (HPSP) were replaced by an integrated logistical system, including unified arrangements for procurement, storage, distribution and transportation. The intention was to improve service quality, motivate employees to perform better and reduce cost. An evaluation of the procurement system reported that quality and motivation problems persisted. Continued drug shortages were attributed to extra bureaucracy in the procurement guidelines, lack of skills in the actual process of procurement, and financial limitations [19].

##### Community clinics

In order to expand access and coverage to the essential service package and to replace labour-intensive and costly outreach family planning services with a cost-effective package at one location, community clinics were constructed for every 6,000 population [20]. However, the persistent unavailability of drugs and supplies reduced the clinics to the provision of family planning and in some cases child immunisation only [21].

#### Uganda

In the years after independence in 1962, the public service of Uganda, including the health service, was regarded as one of the most effective in sub-Saharan Africa. However, health services for the most part collapsed during the 1970s and 1980s [22]. Since then Uganda has implemented a series of reforms, specifically the decentralisation of service delivery and accompanying reforms in civil service which were both aimed at achieving the goal of making the central and local authorities function in an efficient and democratic manner.

##### Decentralisation

Decentralisation of health care delivery in Uganda had a dual context. The first entailed decentralisation of all government services that were delivered by the central government with the aim of devolving power to district authorities. The second context was the transformation of roles in the health sector in response to the decentralisation policy.

Matters relating to personnel were part of the decentralised functions. A new structure, the District Service Commission, was formed at the district level to perform the functions of personnel management, under direct supervision and guidance from the national Public Service Commission and Ministry of Public Service. The main human resource management roles at the district level were to identify staff requirements and their training needs and to ensure that health facilities had the minimum staffing requirements. In addition, the powers to recruit, exercise disciplinary control, promote and to remove persons from district service were delegated to the District Service Commissions (DSCs).

A critical change in the employment system under decentralisation was the demand-driven recruitment into the district service. Each district would advertise the available jobs within its department before any recruitment was undertaken. This was a major shift from the previous centralised system where medical and paramedical professions were recruited by the Ministry of Health (MOH) immediately upon graduation and posted directly to selected workstations.

However, the revenue base of the local authorities was poor and depended on grants from the central government to pay the district personnel. Grants from central government were enshrined in hard budget constraints (conditional grants) that did not provide for local flexibility in resource allocation [23].

##### Civil Service Reforms

The civil service reform (CSR) programme was to address four key areas: personnel management; organisational structure; performance accountability; and service conditions [24].

The excessive size of the Ugandan civil service was understood to explain inefficiency, poor performance, and inadequate pay and benefits. Between 1990 and 1997, the public service was reduced by 54 per cent without a clear agreement on the target-size of staff between the Ministry of Finance and that of Public Services [25]. Further components of the CSR included decentralising power over personnel management to district service commissions, introduction of results-oriented management (ROM) and capacity building [22].

##### Pay reforms

By 1995, after salary increments totalling 85 per cent over 1986, wages and salaries in the civil service were still perceived as being insufficient to maintain an adequate standard of living [25,26]. After 1995, the pay reforms were abandoned in light of deficient revenue collection and alternative demands concerning the initiation of a universal primary education programme.

The first phase of the salary enhancement plans addressed the judiciary and justice sub-sector. This boosted earnings of the entry-level lawyers to a point that was three times that of a consultant medical doctor. The perceived inequity was responsible for several strikes between 1990 and 1995 among medical staff. In response, a lunch allowance for medical workers was introduced in 1994 [27,28]. Furthermore, the government consolidated all allowances, such as housing, medical and transport, into salaries but at the same time imposed a pay-as-you-earn tax (30%) on the consolidated salary; a decision that reduced the overall impact of the strategy to increase earnings. However, the regularity of salary payments improved due to the fact that earmarked finances were being sent to the districts as conditional grants [27].

Due to budget constraints, the civil service recruitment into the district service has been largely frozen since 1993. Although local governments had the authority to recruit, salary delays and arrears in the district payrolls resulted in health workers salaries being reassigned to the central government payroll in 2000 [29].

##### User fees

During the 2001 presidential election campaign, user fees in the public health delivery system were outlawed. An increase in the number of clients seeking services occurred [29] as did stock-outs of drugs and essential supplies. The Ministry of Health increased operational budgets to the health facilities in order to cater for the increased demand for drugs [30]. This, however, did not compensate for the staff's loss of their previous supplementary income through user fee revenues [31,32].

##### Health sub-district

Low accessibility to basic health services had persisted and gaps in staffing especially in the rural health units were attributed to weak management of health services below the district [33,34]. A policy decision was made to further decentralise health service delivery to the county level, corresponding with the political constituency or the "Health Sub-district" (HSD). In line with this process, Health unit management committees (HUMC) were formalised and strengthened to oversee the work of the health facilities and also act as the link with the communities.

Several strategies were adopted to achieve the HSD objectives. These included the upgrading of some health facilities to provide a comprehensive set of essential health services as well as harnessing available hospital capacity for disease prevention and health promotion activities. In addition, the health sub-district personnel were required to provide managerial support to 7–15 lower-level health facilities within their defined geographical area [34]. These additional support roles added a substantial workload related to administration i.e. supervision, financial management, coordination and reporting.

### The human reactions to reforms: dynamic responses

#### Survey findings

Health personnel in both Bangladesh and Uganda responded to a questionnaire covering questions on the effects of reforms regarding issues such as management, supervision, training, promotion, salaries, the availability of drugs and supplies and incentives for remote deployment (see Table 5).

**Table 5:** Percentage of health staff agreeing with statement on reforms

	Bangladesh unification reforms		Uganda decentralisation reforms	
	Health	Family planning	Public	NGO

	% Strongly or partially agreeing			
	
The reforms have increased your chances of being promoted	8	7	50	51
The reforms have increased the chance for you to keep your job	31	64	55	46
The reforms have increased the objectivity of the appraised/performance reports	62	74	74	73
Your salary is always paid promptly	93	59	73	65
Your salary increments in the past couple of years have been satisfactory	47	38	19	19
The reforms have made your job description clear	67	83	60	55
The equipment essential to perform assigned tasks is available in sufficient quantities	75	56	63	58
The drugs and supplies required to accomplish your tasks are always available	70	32	70	65
The reforms have made your workload more manageable	64	83	52	49

The survey data suggest that in Bangladesh the reforms have affected the health staff and former family planning staff in different ways. While family planning staff consider themselves to have least benefited from the reforms concerning their salaries, promptness of pay and the availability of drugs, supplies and equipment, health staff seem less satisfied with their job security and the manageability of their workload. In Uganda, there appears to be less satisfaction with workload and pay, but comparatively more satisfaction with promotion prospects. Generally, both in Uganda and Bangladesh, health staff seem more satisfied with the objectivity of performance reports after reforms.

The next section explores the multiple interactions between the key players affected by the reforms and provides an insight into the positive and negative responses triggered by the changes that took place in Bangladesh and Uganda in the context of the reforms.

#### Interview and focus group findings

##### Bangladesh

In Bangladesh, the changes that had affected the authority structures displayed a multitude of reactions amongst health staff, who experienced the impact of reforms on their management arrangements, performance appraisal systems, promotional opportunities, payment schemes and ultimately on their performance and provision of services. The combination of unification efforts and the adjustments to familiar arrangements resulted in a mixture of responses and interactions between health and former family health staff, their senior level management staff and community members who had been involved in the formation of the community clinics.

###### Management

Respondents reported that the unification at the lower levels (Upazila and below) of the health and family planning programmes and the resulting assignment of administrative authority to personnel in the health track had caused a conflict. Family planning directors and their subordinates at the national and district level lost their authority over staff at lower levels.

The informants at the district management levels in Bangladesh were divided on the effects of the unification reforms at the Upazila level and below. Civil surgeons (managers on the health side of the divide) held optimistic views about the new administrative and authority structures at the Upazila. In contrast, the deputy directors of family planning described how the changes had caused turmoil in the functioning of the programmes. It was reported that the new structures were dysfunctional and had demoralised workers that belonged to the family planning programme. The same split in perceptions regarding the newly unified structures was observed among the managers at the sub-district (Upazila) level. The sub-district managers expressed difficulties in exercising their authority due to the continued, and sometimes perceived as deliberate, interference by higher offices in the functioning and allegiance of sub-district personnel. Informants confirmed that the unification reform eroded the authority of managers:

*"We are supposed to supervise the Health Assistants, Health Inspectors and Family Planning Inspectors but these people do not follow our advice and orders and the boss (UHFPO) does not instruct the persons to cooperate with us" (SACMO and FWV group)*.

Complaints concerning the bureaucratic obstacles in accessing administrative services at the management level were also voiced in the focus group discussions. These included delays in allowance payments and bribes that workers felt obliged to pay to their managers for their allowances to be approved and paid.

"We have faced problems with the three Drawing and Disbursement Officers and more managers. To get the supplies, transport and other allowances and bills paid we have to move to several offices, which hampers our work and causes dissatisfaction" (Senior staff nurses group, Bangladesh)

###### Performance appraisal systems

There had been a dual system of performance appraisal in the unified structure. Historically, the family planning personnel used a service book, which monitored the daily activities undertaken by each worker. Given the prospects for transferring the Family Planning personnel to the revenue payroll, the service book was used as the basis for successful transfer and in addition determined the level of salary to be received upon transfer into the civil service. The administrative arrangements assigned some of the supervisory roles for the health service track personnel to the family planning track officers and vice versa. Discussants expressed suspicions about performance appraisal reports by superiors who belonged to the rival track, claiming Annual Confidential Reports were being used as punishment and as a way to settle scores between personnel and supervisors.

###### Career structure and promotion

Although promotional and career structure problems preceded the reforms, the common perception in Bangladesh suggested that the problems were aggravated or remained unsolved. Unification reforms assigned a critical element towards promotion, that is, annual confidential reports (ACR) from the trusted vertical programme hierarchy to a new set of officers. The competence of the new assessors of performance was contested partly due to organisational mistrust and possibly due to the dismantling of the well-knit network of patronage in the previous structures.

###### Salaries and promptness of pay

Predominant views emerging from the FGDs (focus group discussions) suggest that salaries had not increased appreciably under the unification reforms. Many health workers expressed concerns over their income and mentioned a number of strategies to cope with the insufficiencies. Most strategies related to activities outside their official work including cultivation, drug shops and work at private clinics. Low and unpaid salaries were among the dominant concerns arising from focus group discussions.

*"We can hardly meet expenses for 2 weeks from our salaries. We have to live at a low standard compared to the status we hold. This is degrading" (FPO and MA at sub-district level)*.

Among family planning staff, delays in salary payment were commonplace, prompting enthusiasm for promised transfer to payment from the revenue budget, the same payment scheme that health staff were benefiting from.

*"For six months I have not been paid any salary. My wife asked me if I am working at my job or doing other things" (FWAs & FWI group)*.

*"We are told that 40 per cent of our colleagues have been transferred to the revenue budget and the rest of us will be transferred soon. This will give us an opportunity to earn salary on time" *(Sub Assistant Community Medical Officer & Family Welfare Visitor group, Bangladesh).

Personnel on the established government civil service received their salaries regularly on the first week of the proceeding month while those that remained on the irregular payroll still suffered from delays in their payments.

Respondents in Bangladesh suggested that the changes in allowances had resulted from the scaling down of fieldwork and the introduction of new administrative structures that threatened the established systems to access allowances and challenged the long tradition of networks that the personnel had built over time with their superiors. As mentioned earlier, the distribution of financial control across three different persons after unification introduced tighter micro-bureaucracy and gate-keeping that made access to allowance and claims difficult.

"To process the allowance bills, you go to the Upazila Family Planning Officer and he tells you to go to the Upazila Health and Family Planning Officer (UFHPO). The UFHPO tells you to go back to Upazila Family Planning Officer and so on. The bill is not paid for one month ... you have to travel to see what has passed. When it is passed one has to pay commission to the bosses and to the clerks. It is frustrating" (Family Planning Inspector and Family Welfare Assistant group, Bangladesh)

###### Community expectations

In Bangladesh, community participation was demonstrated by donation of a plot of land, construction of buildings to house the clinics and the formation of community clinic groups (CCG) for clinic management. Initially most health workers applauded community involvement, especially because it encouraged learning about the constraints under which they were working. During the interviews and focus group discussions, it became clear that the health workers were aware of how the changes in their performance and the effects on service provision were perceived by the communities.

i. Availability of drugs and supplies

One of the stated objectives of the health sector reform was to ensure that the procurement and supply of drugs to the community clinics was brought under unified management and made more efficient in terms of cost reduction and saving time, thus ensuring steady and better service delivery. Discussants stated that this objective had not been reached and claimed that they were thus unable to adequately perform their tasks due to the irregularity in the provision of supplies. They further described how procurement problems caused anger and incomprehension among communities that initially had welcomed the community clinics:

*"There was enthusiasm among the community people and their views about the Community Clinics were positive ... because distances for minor illnesses were reduced. People were competing to provide land for the clinics but when the drug supplies stopped, the people could not see the benefits ... their views are now unfriendly" (Upazila Health and Family Planning & Medical Officer group, Bangladesh)*.

*"It is not serious to go to the clinic daily and sit there doing no work. People come for family planning or cough and you tell them there are no medicines. It is risky... some time they abuse us and complain why we are being paid salary" (Sub Assistant Community Medical Officer, Family Welfare Visitor group, Bangladesh)*.

ii. Adequate service provision

In Bangladesh the service integration between family planning and health services brought a new set of tasks to be carried out by all frontline personnel. In effect, the integration added several services, for example, child immunisation, basic treatment of ailments and directly observed therapy (DOTS) to the family planning personnel. Views emerged during the discussions with health staff suggesting that family planning activities had been displaced by their new tasks under integration, at Upazila level. Many participants indicated that the increase in workload left less time for patients with family planning needs:

*"I am required to provide services at the clinics and visit households for Family Planning. It is impossible to do all this work. Family Planning suffers because there are many Expanded Programme of Immunisation (EPI) clients to see at the clinic" (FPI and FWA group, Bangladesh)*.

*"At the inauguration of the CC we were supplied drugs once or twice and the community response was good. But there are fewer persons attending at our units [union level].... the clinics have not received any supplies now six months after their [official] opening" (group discussant SACMO and FW, Bangladesh)*.

##### Uganda

In the Ugandan context, the decentralisation efforts brought about a rapprochement of local authorities, district boards and health workers who previously had enjoyed relative autonomy from local management structures. Here the closer contact and growing relationships with local authorities together with the changes in accountability structures brought about a multitude of responses amongst health workers, specifically concerning their recruitment patterns, job security, supervision and relationship with community members.

###### Recruitment

The new authority structures that came with the decentralisation policy were the district councils, the district service commissions and the health unit management committees. In the discussions, health workers had differing opinions regarding the influence and newly acquired decision-making powers of these structures regarding specifically the issues of recruitment and supervision.

"Recruitment by the district is now faster and makes it possible to get quickly on the payroll ... this was taking years before" (District Director of health services, Uganda)

"When you go for the interview, these people (district service councillors) interview you in the local language and use complex parables and proverbs to fail you. If you do not understand the questions you are technically out" (Group Discussant, Clinical officers, Uganda)

"...some few, especially sons and daughters of those in positions of influence in the district don't bother with the recruitment process. They are just appointed." (Focus Group Participant, Uganda))

Personnel working away from home viewed the process of recruitment as inward looking and biased against workers from districts that were not home to the new authorities. Local informants explained that the expression "sons and daughters of the soil" is widely understood to describe the phenomenon of preference for workers who originate from the district. While territorial patronage systems had already been observed before reforms, respondents claimed nepotism was present to a higher degree with the relocation of decision-making authority to local bodies under decentralisation.

###### Job Security

In Uganda, health workers with management responsibilities expressed fears about their precarious relationships with the local authorities. They claimed that their concerns mainly arose from local authorities' decision-making power in the dismissal of health workers. The views below also point towards a patronage network that is claimed to have strengthened under decentralisation.

"...For some of us who work in rural places if you disagree with your sub-county bosses on any matter, that boss will automatically make sure that he at least punishes you and you lose your job" (FGD Midwife, Uganda)

*"I think the relationship is good. If you give them [district counsellors] what they want, they also make our work easy. .. Sometimes they take the only vehicle for burials or campaigns and we hold back the activities. At the end of the day one has to be in good books or you are thrown out" (Assistant District Health Director, Uganda)*.

###### Supervision

In addition to the structural changes that influenced recruitment patterns and job security, the type and focus of supervising the health workers changed with the new structures in Uganda. It was reported that HUMC, CAO (Chief Administration Officer), DSC and local politicians were increasingly undertaking the supervision of personnel and health facilities. Health staff described how before decentralisation, supervision was geared more towards the technical quality of workers encouraging them to undertake new service roles or to improve their services. According to their accounts after the reforms, supervision took on a more human angle with the direct accountability of health staff to selected community members. In general, health staff seemed to appreciate the new changes in their supervision since relationships to key community members improved through tighter working relationships.

"Decentralisation has fostered better supervision of facilities by the district because they are now answerable to the people at the district or within their areas of jurisdiction" (Secretary District Service Commission, Uganda)

"... The health unit management committee has come to realize that the major problem is not selling of drugs but the small quantities always supplied. Now the relationship is not so bad as it was in the past" (Focus group discussant, clinical Officers, Uganda)

Views also emerged from the focus group discussions in Uganda that suggested the health staffs' relationships specifically with community leaders had changed with decentralisation. There seemed to be an overall improvement.

"Leaders now appreciate the work we do and are keen to support us. ... they have been articulating our needs for more staff at the district council" (Key Informant Interview, Facility In-charge)

Despite several attitudes describing the positive relationships and increased understanding between local authorities and health staff through the changes in supervision, several statements displayed the workforce's concern about relationships with those community members that were less involved in overseeing the provision of health services.

###### Community expectations

Communities expected the quality of services to improve markedly when user fees were removed as had been promised during the presidential election campaigns. The increase in the utilisation of health services after the removal of fees put pressure on the availability of drugs and supplies leading to marked shortages. Health workers reported a sense of feeling caught in the middle of the financial shortcomings of government and the high expectations created by the publicity about the removal of user fees.

*"How do you expect us to handle that problem (too many patients and too few drugs)? We used to buy drugs from the fees but now we are just spectators. We tell them OS (out of stock) and they say we are not kind to them" (Group Discussant, Clinical Officers, Uganda)*.

Staff in Uganda described how communities were surprised when medical supplies and drugs would suddenly run out in times of need. In fact, FGD discussants reported how patients would question the trustworthiness of health staff in the facilities.

"There has been a concept... that medical people steal drugs from health units. Drugs kits would be delivered and three days later we would tell them (patients) that the drugs are finished. They did not believe ...they would say that we have stolen the drugs..." (Group Discussant, Clinical Officers, Uganda)

While the quantitative data provide a general picture of staff attitudes and their responses to the reforms in the two countries, the qualitative data explore how reforms have been interpreted and perceived, and how dynamics between key players reveal whether or not the workforce is likely to consist of active and enthusiastic implementers of reform or dissenters, actively or passively resisting implementation. The findings from the qualitative research suggest less satisfaction with the changes that had taken place with the reform initiatives compared to the survey results. This could be due to the nature of the methods, if people are less likely to answer negatively when prompted with a questionnaire than in focus groups or interviews, using open-ended questions.

## Discussion

This paper has presented findings from a comparative analysis of two country case studies investigating the impact of health sector reforms on human resources in Bangladesh and Uganda. Pawson and Tilley's [13] proposition for realist explanation provided a useful framework for the exploration of the mechanisms by which health sector reforms affect health workers' micro environments, thus changing provider incentives and creating a multitude of responses. By using the dynamic responses model for health system research, this paper provided an insight into the de-jure system from which the reform objectives were planned and initiated and traced the resulting dynamic responses between different levels and types of health staff and the communities.

In both Uganda and Bangladesh, reform planners neglected the role of context in their planning of reform objectives and assumed that the workforce would act as a passive element in the reform implementation. In this sense, the study highlighted several issues in both countries that demonstrate the importance of careful analysis of contextual factors in the design and implementation of reform objectives and the significance of recognising the workforce as an important and adaptive factor contributing to the success or failure of the reforms.

In Bangladesh, the effects of reforms resulted in a power struggle and general mistrust between the family planning and health tracks. A strong sense of inequity in the nature of effects of the reform was illustrated by family planning personnel who perceived they had lost out while health track personnel had gained from the reforms both in terms of authority and actual reduction of tasks under integrated provision. There remained considerable distrust within the new unified structure, with reluctance to take orders from the new managers. The non-unified top administration above the integrated Upazila level was perceived as a potential source of conflict and ill intent. In addition, drug shortages and general procurement failures emerged as a source of bad public relations between the workforce and the communities despite originating in broader systemic problems such as financing and budget constraints that had already existed before the initiation of reform objectives. The communities participated in the planning process and priority setting through community structures, however, their decisions were not binding since the Ministries of Finance imposed budget cuts and ceilings motivated by broader pressures in the economic context.

On the other hand, positive responses from the majority of former family planning staff expressed hopefulness regarding the changes that would secure salary payments and inequities in schemes and demonstrated optimism concerning prompter salary payments under the revenue budget. This suggests a rise in motivation levels in balance with the right incentives.

Ugandan findings show how the workforce responded to a strong and rapidly implemented system of decentralisation that had insufficient competence for human resource management. Power of local authorities was influenced by resource constraints and nepotism in recruitment. Subsequently, these constraints suppressed the incentives of workforce related to promotion, job security and professional growth. Health workers were more insecure as a result of decentralisation due to their dependence on the benevolence of the new authorities in order to guarantee their jobs.

However, closer ties with selected community members also had positive effects for health workers. It was suggested that community leaders in their new supervisory function were able to witness the precarious position that health workers had as middlemen between budget constraints and procurement failures at higher levels of the system and the communities who expected smooth running services and consistent availability of drugs and equipment. In this sense, local leaders with their newly acquired authority were able to lobby for necessary financial and human resources for the efficient operation of the health centres.

While the de-facto system has not specifically been addressed in this paper, the outcome of the relationship between the de-jure system and the dynamic responses for those that ultimately use the services are varying. While users on the one hand may be confronted with demoralised and in some cases absent staff, lack of necessary drugs and equipment and dilapidated health facilities, the shift of authority and supervisory functions towards the rural districts, on the other hand, seems to have positively affected communities who are now actively involved in the shaping of their health service provision with their newly acquired responsibilities.

## Conclusion

This paper has emphasised several key points that are summarised below:

1. By keeping the dynamic responses model in mind, national and international reform planners can design reform objectives that ultimately enhance and improve services as felt by the communities by encouraging favourable responses amongst the workforce.

2. Reform planners need to take a closer look at the context within which the health system operates in order to recognise potential 'inhospitable elements' which may hinder reform objectives or 'hospitable' elements which may support reform initiatives and provide a basis for improvements in the operation and management of health systems.

3. Reform programs need to incorporate active implementation research systems to learn the contextual dynamics and responses, as well as have inbuilt program capacity for corrective measures.

4. Health workers are key stakeholders in any reform process and should participate at all stages, that is, conceptualisation, design and implementation. Reforms tend to create losers and winners or can change power structures but it is important, at the least, that winners and losers understand the purpose of change and have confidence in the process of consultations on which change has been determined.

5. How health workers perceive their relationship with the community will affect their job motivation and performance. This is an important but neglected criterion for evaluating the impact of human sector reforms.

## Competing interests

The author(s) declare that they have no competing interests.

## Authors' contributions

CH, FS, BM were involved in the development of the protocol. FS and ER carried out the fieldwork in Uganda and SAR and AM in Bangladesh. FS, SAR, ER, AM, CH, BM and TK undertook the analysis of the data and TK, BM, FS, SAR and ER drafted the manuscript.
